# The Prevention of Consumption

**Published:** 1905-10-28

**Authors:** J. W. Miller

**Affiliations:** Assistant Medical Officer of Health, Blackburn


### Hospital Clinics.

### THE PREVENTION OF CONSUMPTION.

By J. W. Miller, M.D., D.P.H., Assistant Medical Officer of Health, Blackburn.

(*Continued from page 48.*)

For treatment consumptives can be divided into two classes:—

1. Early cases.
2. Advanced cases.

### Sanatorium Treatment.

So far there can be no doubt that this treatment has produced the best results for early cases and in some cases effected a complete cure.

In every county in England and Wales sanatoria should be provided by each county council. In some counties sanatoria are already in process of erection, whilst in other counties such institutions are being contemplated. In some of the larger towns sanatoria should be provided by the sanitary authority.

Cases could then be drafted from the towns to the sanatoria, and in the case of the sanatoria under the county council a contribution could be made by the urban council for each case, or a certain number of beds could be retained and a yearly amount paid to cover the cost of these beds. In addition cases from rural districts would need to be provided for. Co-operation between the various friendly societies is also necessary for the erection and maintenance of sanatoria.

There is no necessity for the erection of palatial and extensive buildings, as the open-air treatment can be carried on in shelters erected at a very moderate cost. The majority of consumptives, if taken in an early stage, improve under sanatorium treatment and in some cases permanent cures are effected if the treatment is continued for a sufficient length of time and the open-air treatment and feeding are kept up when such persons return home. In a good many cases the improvement which has taken place is nullified by the conditions under which such persons live on their return home. In many cases the additional nourishment required cannot be kept up on account of expense, and the open-air treatment is not persevered with; in addition, although improvement has taken place, indoor occupation cannot be followed.

For such persons some institution is necessary where treatment can be kept up and light work undertaken.

In regard to persons who return to an outdoor occupation, if open-air treatment is persevered with and additional nourishment taken, there is no need for continued treatment in such an institution.

### Colonies for Consumptives.

These would provide for those consumptives who have improved under sanatorium treatment but are not yet fit to follow their ordinary occupation. Difficulties are met in obtaining the requisite amount of fresh air required either in the office, workshop, or in the home. Draughts are complained of by relations and fellow workers if windows be widely opened all the year round. Many consumptives on return home from a sanatorium are able to do light work, but, unfortunately, this means less money available for suitable house accommodation and the additional nourishment required. These cases should be sent to a colony for consumptives where the open-air treatment can be continued and light work undertaken, preferably outdoor, but in some cases indoor.

At the Victoria Dispensary for Consumptives, in Edinburgh, the various duties of porter, gardener, etc., are undertaken by the consumptives themselves and the cost of administration is considerably reduced in consequence.

Such colonies could be maintained by the county councils, also by the sanitary authorities of the larger towns.

### Reception Hospital for Advanced Cases.

Advanced cases of consumption need to be dealt with as they are the most potent means for the spread of the disease whether at home or in the office and workshop. These cases need removing from surroundings which in many cases are only too favourable for the spread of the disease.

A few of these cases find their way into the workhouse hospital, but the majority prefer to remain at home to drag on their miserable existence under the most unfavourable conditions.

Some provision should be made by each county council for a reception hospital at which such cases can be received, whilst under certain circumstances such a hospital could be maintained by the urban or rural sanitary authority.

All cases would be known to the health authorities through notification; the ordinary notification forms for infectious disease could be used, and in addition the following information asked for:—

1. The occupation of the patient, if any, and where this occupation was followed—*e.g.* in the case of a weaver the name of the mill would be given.
2. The duration of the disease.
3. The suitability of the case for

   *a* Sanatorium treatment,
   *b* A colony for consumptives,
   *c* A reception hospital.

The above particulars could be given in all cases, whether the patients were attended by their own medical attendant, or whether treated in the out-patients' department of a general hospital, hospital for consumption, or dispensary.

In the case of consumptives attending a general hospital, provision should be made for rooms built away from the out-patient department and which patients could reach without passing through any other part of the hospital; this would insure freedom from infection to the other out-patients.

Cases attending the out-patients' department and found to be affected with the disease could be directed to this special department for consumptives.

In some of the larger towns a dispensary for consumptives would be a useful institution, as all persons affected with the disease could attend at this dispensary instead of visiting the various general hospitals.

Cases diagnosed at the general hospital could be directed here.

At the Victoria Dispensary for Consumptives, at Edinburgh, consumptives are given advice and medicine at the dispensary, and in addition special visits are made in certain cases and advice given in regard to open-air treatment, feeding, etc., carried on at home. This is especially valuable in the case of consumptives who have returned home from sanatorium treatment, as such cases speedily lapse if the open-air treatment is not continued.

All arrangements regarding isolation, disinfection, directions in regard to destruction of sputum and inquiries in regard to occupation and anything that will throw any light on the cause of the disease should be carried out by the health authorities.

Medical practitioners should be asked to inform the health authorities either (*a*) when a patient gives up treatment, (*b*) when a patient removes to another part of the town.

By means of the above information supplied by medical practitioners and special inquiries made, a list could be drawn up and kept at the health office, containing the names of all consumptives and whether suitable for sanatorium treatment or for removal to a reception hospital.

As vacancies occurred in these institutions the most urgent cases could be sent.

There can be no doubt that if the above energetic measures were carried a still greater reduction would be brought about in both the incidence and mortality of the disease; whilst improved sanitation, the teaching of hygiene in schools, would all tend in the same direction.

